# Improving the ‘Golden Patient’ Initiative at a British Major Trauma Centre: A Single-Centre Study

**DOI:** 10.7759/cureus.68918

**Published:** 2024-09-08

**Authors:** Samuel Thompson, Shafiq Shahban, Sanjeev Patil

**Affiliations:** 1 Intensive Care Unit, Northern Beaches Hospital, Sydney, AUS; 2 Department of Trauma and Orthopaedics, Walsall Manor Hospital, Walsall, GBR; 3 Department of Orthopaedic Surgery, Queen Elizabeth University Hospital, Glasgow, GBR

**Keywords:** trauma and orthopedics, trauma patients, golden patient, theatre productivity, theatre efficiency, global healthcare systems

## Abstract

Introduction

Delays in theatre start times are expensive and associated with poor outcomes. To reduce these delays, a Golden Patient (GP) protocol was used at one of Britain’s major trauma centres, the Queen Elizabeth University Hospital, Glasgow. We sought to clarify how often Golden Patients (GPs) were stepped down from being first on the day’s trauma list and to identify significant contributing factors.

Methods

We collected data over an eight-week period, with 80 GPs collated in total. If stepped down, we recorded their age, gender, injury, location, and day of planned surgery. Univariate analyses were then performed to test for statistical significance. We also followed stepped-down patients, noting how long until they received their operation.

Results

The incidence of GPs stepped down from being first on the list was 11.25%. This did not vary with age, gender, or type of injury, but was significantly associated with patients being at home the night before their planned operation (p=0.0114) and cases occurring on Fridays (p=0.0139). Of those stepped-down GPs who remained for operative management, all received their operation within one day.

Conclusion

This study, the first of its kind since the COVID-19 pandemic, shows low rates of GP step down, comparable to previous audits of GP initiatives in similar centres. When delays did occur, GPs received timely operative management once underlying issues were resolved. This study suggests that planned GPs should be admitted the night before their operation. Whilst the GP system serves trauma patients well, we identified areas for improvement in the efficiency of our own service applicable to other busy major trauma centres.

## Introduction

Recently, the coronavirus disease 2019 (COVID-19) pandemic has put the whole of the British National Health Service (NHS) under considerable pressure [1]. During this time, surgical specialties have been managing reduced staffing and ever-increasing waitlists [2]. As such, it is vital to identify and reduce sources of inefficiency within the surgical practice and maximise available resources to improve patient outcomes.

It is well-established that delays in theatre start times are financially expensive, costing approximately 1000 GBP per theatre per day [3]. In addition, these delays are significantly associated with prolonged inpatient stays and increased post-operative morbidity and mortality [4,5]. Furthermore, it has been shown that when the first case of the day is delayed, each later case on that operative list is likely to experience additional, individual delays [6].

The Golden Patient initiative sought to tackle this issue by pre-selecting the first patient for each of the following day’s trauma lists [7]. Termed ‘Golden Patients’ (GPs), these were medically suitable patients with a clear surgical plan, agreed on by the surgical team the night prior to their operation. These would only be stepped down if a life- or limb-threatening case was admitted overnight, requiring prioritisation over the GP. This initiative led to a significant reduction in delays in theatre start time, by an average of 30 minutes, and has been subsequently validated across different orthopaedic departments [8,9] and other surgical specialties [10]. Furthermore, a recent systematic review demonstrated that departments that implement a GP initiative consistently benefit from improvements across theatre arrival, anaesthetic, and procedural start times [11]. We hypothesise that stepping down GPs could lead to delays in theatre start times, which are known to correlate to worse post-operative outcomes [4,5]. This highlights the importance of identifying any potential underlying causes of GP step down, which might then be addressed, potentially improving patients' experiences and outcomes. 

The Queen Elizabeth University Hospital Glasgow is a large teaching hospital and major trauma centre, treating over 1000 critically and severely injured patients per year [12]. As our Trauma and Orthopaedics department already operated a GP system, we sought to improve theatre efficiency and patient outcomes by identifying how often any pre-determined GPs were stepped down from being first on their theatre list, and whether there were any significant underlying factors that contributed to this occurring.

## Materials and methods

Data were collected during an eight-week period between December 2021 and February 2022. We attended morning trauma meetings, where patients on the trauma board were discussed and theatre lists finalised for the day ahead. All GPs discussed in the meetings were considered eligible for inclusion. We recorded the GPs’ age, gender, injury, and location within the hospital. Also noted was the day of the week that the trauma meeting occurred. Recorded data were promptly stored in an Excel document (Microsoft Corporation, Redmond, USA) on a password-protected, limited-access drive on the secure hospital network, ensuring accuracy of results and alignment with information governance protocols. 

The main outcome of interest was whether each GP remained first on the list for that day, or whether they were stepped down, with another case taking their place. In instances where this occurred, we noted the underlying reasons for this, and whether the decision was made by the orthopaedic or anaesthetic team. We also followed up with the subset of GPs who were stepped down, looking at whether these patients ultimately underwent their planned operation, and if so, how long their case was delayed.

We then performed a univariate analysis of the relationships between the GP characteristics, and whether GPs remained first on their theatre list following the trauma meeting. This used either Fisher’s exact test (categorical variables) or the Mann-Whitney U test (continuous variables). All tests were two-tailed, and the significance was set as p<0.05. All statistical analyses were carried out with RStudio 2022.2.0.443 (RStudio Inc., Boston, MA, USA). 

## Results

Eighty GPs were identified from attended trauma meetings. Nine patients were stepped down due to no longer being first on the list (11.25%). The risk of a GP being stepped down was not affected by their gender, age, or injury, but was significantly associated with being at home on the day of their case (P=0.0114) (Table 1). In addition, GPs discussed at a Friday trauma meeting had a significantly higher risk of being stepped down (P=0.0139).

**Table 1:** Golden Patient Characteristics IQR: Interquartile Range. NOF: Neck of femur fracture. Data are number (%) unless stated otherwise. a = Fischer’s exact test, b = Mann-Whitney U test (the U value for the Media Age comparison was 333). P values denote the results of univariate analyses comparing those who remained first on the theatre list, to those who were stepped down by the end of the trauma meeting. Bold P values indicate statistical significance.

		Golden Patients (n=80)		
		Remained first on list (n=71)	Stepped down (n=9)	P
Gender	Male	33 (46%)	5 (56%)	0.7292 ^a^
	Female	38 (54)	4 (44)	
Median age (IQR)		63.00 (48.00, 75.50)	61.00 (50.00, 79.00)	0.8431^ b^
Injury	Upper limb	22 (31)	3 (33)	0.7453 ^a^
	Lower limb (non-NOF)	23 (32)	4 (44)	
	NOF	16 (23)	1 (11)	
	Pelvic	6 (8)	0 (0)	
	Polytrauma	4 (6)	1 (11)	
Location	Inpatient	71 (100)	7 (77)	0.0114 ^a^
	At home	0 (0)	2 (22)	
Day of case	Monday	12 (17)	0 (0)	0.0139 ^a^
	Tuesday	13 (18)	3 (33)	
	Wednesday	15 (21)	1 (11)	
	Thursday	20 (28)	0 (0)	
	Friday	11 (15)	5 (55)	

Of the nine patients who were no longer GPs by the end of the trauma meeting, seven were stepped down by the orthopaedic team (Table 2). Of these, two had originally been at home and subsequently did not attend the admissions ward on time. The first of these was known to have anxiety surrounding his operation and did not attend the ward at all. The second was operated on later that day once they attended the ward.

**Table 2:** Reasons for Golden Patient Step-down AWI: Adults With Incapacity. CT: Computerised Topography. INR: International Normalised Ratio.

Case	Reason for Step-Down	Decision	Result
1	Did not attend ward on-time (severe anxiety)	Orthopaedic	Conservative management
2	For conservative management (patient’s preference)	Orthopaedic	Conservative management
3	For conservative management	Orthopaedic	Conservative management
4	Low haemoglobin despite administration of blood products	Anaesthetic	Closed, rather than open, reduction of dislocation
5	AWI not completed	Orthopaedic	Same-day surgery
6	Further examination of patient by operative team required	Orthopaedic	Same-day surgery
7	Fall overnight, subsequent confusion and required CT imaging	Anaesthetic	Same-day surgery
8	Did not attend ward on-time	Orthopaedic	Same-day surgery
9	High INR in a warfarinised patient	Orthopaedic	Next-day surgery

Two more previous GPs were deemed to be for conservative, rather than operative, management, and one required a further assessment by the subspecialty operative team prior to being taken to theatre. A further GP who was intubated in the ICU (Intensive Care Unit) had their case delayed as they were unable to directly consent for the operation and the appropriate Adults with Incapacity (AWI) form had not been completed by the time of the trauma meeting. The final orthopaedic reason for the delay was for a patient on warfarin, whose International Normalised Ratio (INR) remained overly high on morning bloods despite receiving intravenous vitamin K overnight. 

Two GPs were stepped down during the trauma meeting by the anaesthetic team. One had a haemoglobin of 85 g/L, which was deemed too low for the planned operative intervention. The second patient had fallen overnight and required a CT head to investigate for intracranial injury prior to theatre. 

For the nine GPs stepped down, all who remained for operative management had their operations either later the same day or the following day (Table 2). 

## Discussion

To our knowledge, this is the first study exploring the underlying causes of GP step down in an orthopaedic department that already utilised the GP system and the first examination of the GP initiative since the COVID-19 pandemic. In the original demonstration of the GP initiative, Javed et al. found that 88% of GPs remained first on the list by the end of their trauma meeting [7], comparable to the 89% we found in our study. Delays to GP surgery due to life- or limb-threatening admissions following a finalised list have been previously reported [9] but were not observed in this study. 

Across our GPs, age, gender, and type of injury were not associated with a greater risk of step down. This implies that there was no measured demographic of trauma patient who was being poorly served by the GP system. There was however a significant association with patients being at home on the morning of their planned surgery (p=0.0114), with these two GPs either attending the ward either too late or not at all. This reflects the findings of Tulloch et al., who saw all three of their neurosurgical GPs who were planned to be admitted the morning of their surgery, rather than being inpatients overnight, experience delayed theatre start times [10]. Therefore, even though demand for inpatient beds in health systems grappling with post-COVID-19 pandemic pressures may be considerable [1], efforts to conserve resources by having GPs stay at home the night before their surgeries will likely lead to stepping down that GP, thus unintentionally triggering the additional costs of a delayed theatre start time [3]. Ultimately, this cross-specialty agreement highlights that patients assigned first-on-the-list status should be admitted to the hospital overnight to avoid such delays. 

The risk of delay also varied significantly by day of the week (p=0.0139). Over half of the GPs stepped down were listed for a Friday. In our department, this is the day that the on-call trauma team changes. Of note, only three of these five were stepped down by the orthopaedic team, with the anaesthetic team deciding the remaining two were not suitable for theatre. Whilst clear communication at times of handover is vital, we would need a larger dataset before the relevance of this result could be meaningfully commented upon. 

It is of interest that two stepped-down GPs had a subsequently documented preference for conservative management. Whilst they had both been initially consented to and were happy to proceed with operative management, consent is a dynamic process that, when possible, patients should control at all stages pre-surgery [13]. As such, it could be argued that patients should not be made GPs when there is a suggestion that they may have doubts regarding the operative management of their condition. 

Four of the GPs stepped down in our study had issues that required resolution prior to proceeding with surgery, including further assessment by a subspeciality team for a complex operation, an incomplete AWI, pre-operative anaemia, and raised INR. In Scotland, having an appropriate section 47 documentation for a patient who is unable to consent to a procedure is established best practice [14]. Interestingly, the single incident where a patient lacked a valid AWI form occurred for a patient intubated in the ICU, which may be why this was missed. Pre-operative anaemia is associated with worse patient outcomes [15]­­, with guidelines acknowledging the advantages of transfusing to a target haemoglobin [16]. The GP in this study who was stepped down due to pre-operative anaemia had been transfused overnight, but the morning haemoglobin level was not felt to be adequate to proceed with the planned operation. Similarly, the case of raised INR was delayed despite overnight treatment with phytomenadione as per local guidelines. It could be that rates of GP step down may be reduced by avoiding assigning GP status to patients requiring further pre-operative optimisation overnight. 

We note that we would not have identified cases where GPs who were not stepped down had similar medical issues successfully managed overnight, which could potentially give us a selection bias. Other limitations of this study include a limited timeframe of analysis and a small sample size, which could leave the study underpowered to detect important clinical findings. In addition, data were collected only on weekdays despite trauma lists with GPs continuing to run on weekends, which may have had different patterns of GP step down. Future reviews with larger cohorts will be important to further explore factors contributing to GP step down, including weekend patterns and whether the probability of GP step down is increased on the day of team handover. 

## Conclusions

Ultimately, across a busy trauma and orthopaedic department, we found a successful implementation of the GP initiative. Some areas for improvement were identified that may benefit comparable trauma services, particularly avoiding assigning first-on-the-list status to patients who were not in the hospital overnight and ensuring appropriate documentation of consent in place for patients in the ICU. There is also potential scope to reduce GP step down by ensuring that patients requiring ongoing medical management overnight are not first on the list. Further research into this will enable our own, and other, busy Major Trauma Centres to reduce rates of GP step down, improve theatre start times, and optimise outcomes for trauma patients.
